# Evaluation and Management of an Uncommon Tumor of the Larynx: A Case Report and Literature Review of Laryngeal Low-Grade Myofibroblastic Sarcoma

**DOI:** 10.7759/cureus.11072

**Published:** 2020-10-21

**Authors:** Nithin P Nair, Darwin Kaushal, Meenakshi Rao, Kapil Soni, Sameema Vaithankalath

**Affiliations:** 1 Otorhinolaryngology, All India Institute of Medical Sciences, Jodhpur, IND; 2 Pathology and Lab Medicine, All India Institute of Medical Sciences, Jodhpur, IND

**Keywords:** larynx, myofibroblastic sarcoma, laryngectomy, tracheostomy, smooth muscle antigen

## Abstract

Low-grade myofibroblastic sarcoma (LGMS) of the larynx is an uncommon entity. These mesenchymal tumors of the larynx are rare and account for approximately 0.3% to 1.0% of all neoplasms at this site. We report a rare case of LGMS of the larynx that involved the larynx of a 63-year-old man with a history of hoarseness of voice. The patient was treated with total laryngectomy with partial pharyngectomy without any adjuvant treatment. Histopathologically, the tumor was composed of spindle cells that manifested variable cellular anaplasia and expressed smooth muscle actin (SMA). Our patient is disease-free two years after surgery. The authors emphasize the clinical and histopathological findings and treatment of this case with a literature review. This case is among the few reported cases of LGMS of the larynx. It enlightens the classical clinical findings, histopathology, differential diagnosis, and treatment. Surgical excision with negative margins requires no adjuvant therapy.

## Introduction

The majority of the laryngeal cancers (85%-90%) are squamous cell carcinomas that arise from the laryngeal epithelial lining. Primary non-squamous tumors of the larynx are composed of approximately 0.3%-1% of all laryngeal tumors [1]. These tumors mainly derive from myofibroblast mesenchymal cells, mostly malignant and rarely benign. Low-grade myofibroblastic sarcoma is a rare atypical malignant myofibroblastic tumor. These tumors have a propensity for local recurrence and rarely metastasize [2]. It is more commonly seen in adult patients, with a slight male predominance. It is often seen in the tongue and oral cavity but seldom seen in the salivary gland, pyriform fossa, nasal cavity, paranasal sinuses, mandible, and parapharyngeal space [3]. To date, there are only 13 cases affecting the larynx reported in the literature [4-5]. Here, we report a case of low-grade myofibroblastic sarcoma of the larynx.

## Case presentation

A 63-year-old gentleman presented to our outpatient department, with a one-year history of change in voice. This history was associated with difficulty in swallowing for six months. He complained of difficulty in breathing for three months for which he was tracheostomised. The patient is a known smoker and tobacco chewer with no other comorbidity.

**Figure 1:**
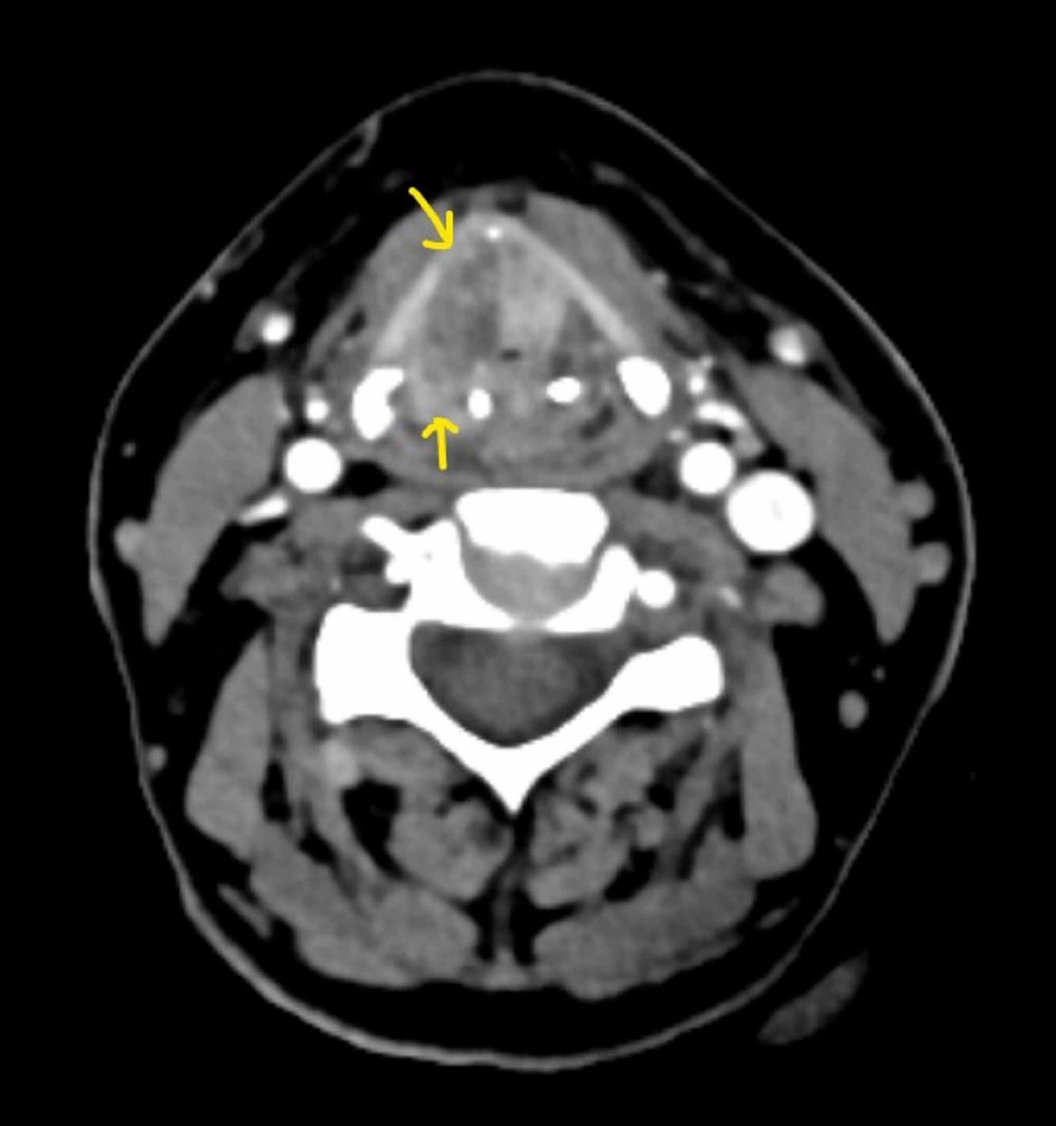
Radiological image (axial cuts) Axial cut, soft tissue window contrast computed tomography of the neck showing a heterogeneously enhancing lesion of both sides of the supraglottis extending to the right pyriform sinus (lower arrow) invading the thyroid cartilage (upper arrow).

On examination, there was no palpable neck swelling. Laryngeal crepitus was absent. Indirect laryngoscopy revealed ulcerative growth involving both true vocal cords, anterior commissure, and posterior commissure extending to both false cords, right pyriform sinus, and subglottis. Contrast-enhanced computed tomography showed a laryngeal mass involvement of both sides of the supraglottis, right pyriform sinus, and subglottis (Figure 1).

**Figure 2:**
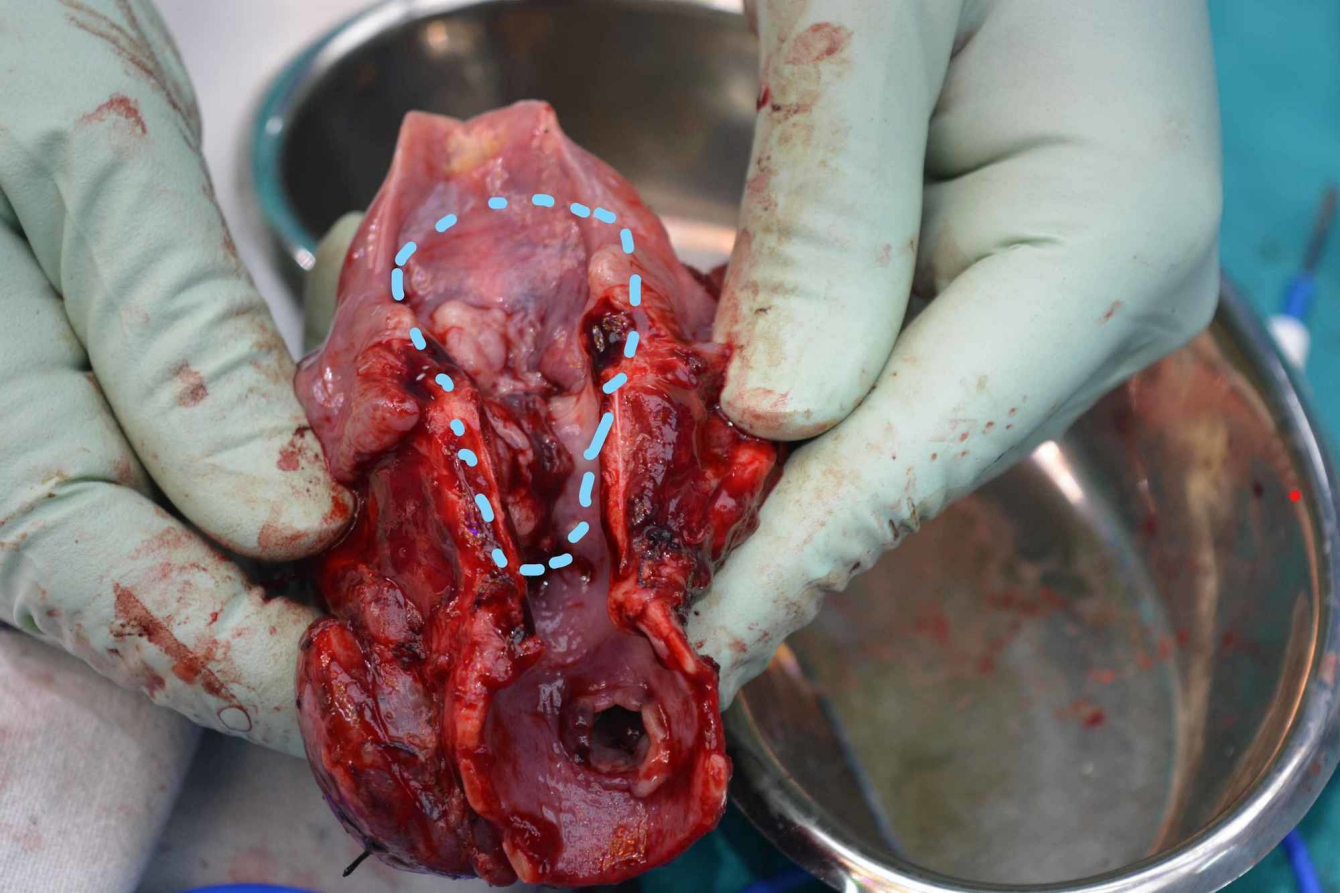
Gross specimen Gross specimen of post-total laryngectomy, partial pharyngectomy, and total thyroidectomy, showing the tumor extending from the supraglottis to the subglottis

The lesion was invading the thyroid cartilage and thyroid gland. The incisional biopsy was suggestive of low-grade myofibroblastic sarcoma. The patient underwent total laryngectomy with total thyroidectomy and partial pharyngectomy (Figure 2). The strap muscles were free of tumor. Primary reconstruction of the pharynx was done.

**Figure 3:**
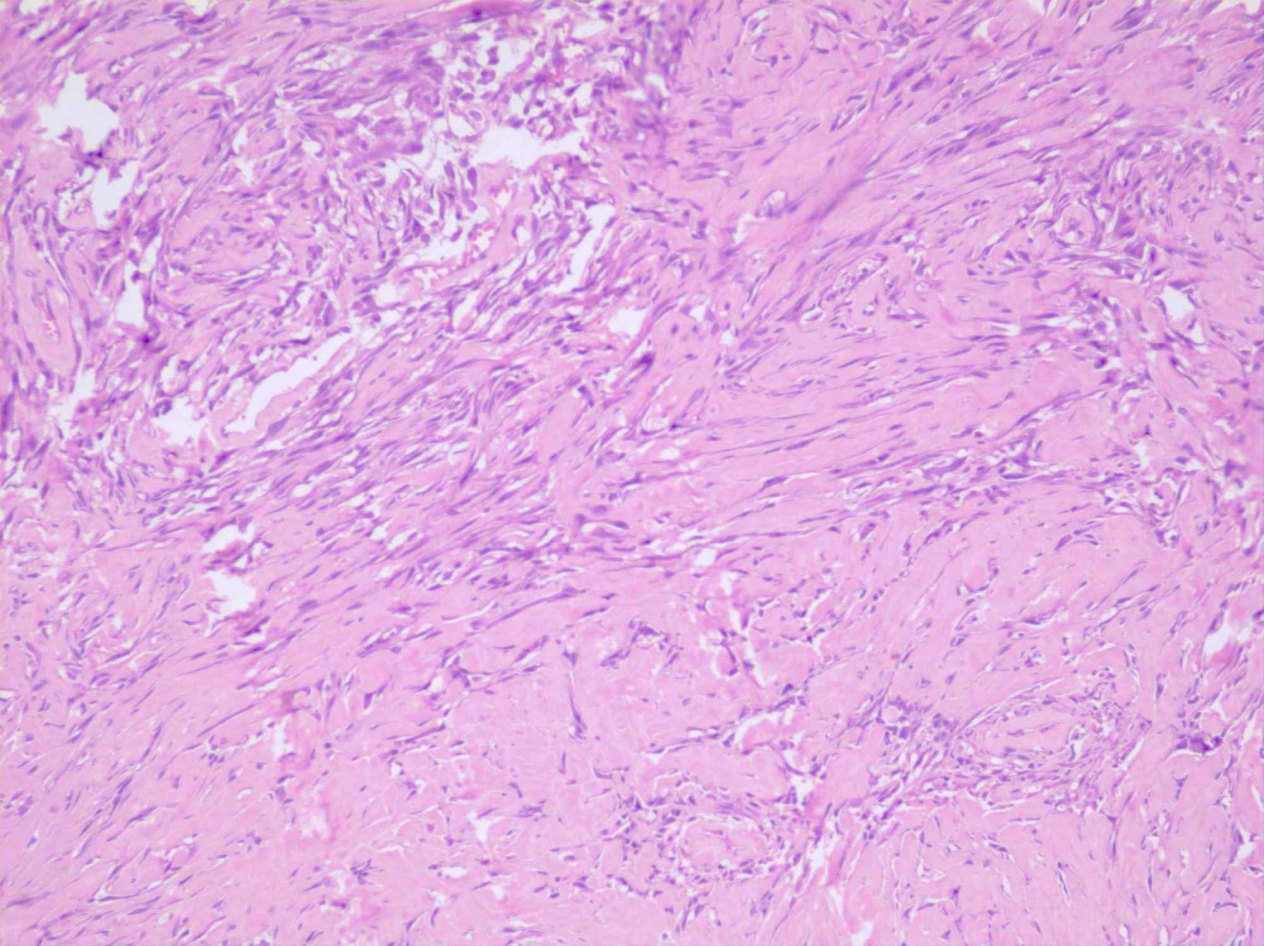
Histopathological examination low power An area of the tumor showing diffuse collagenization, imparting a hypocellular appearance to the tumor (H&E, 10X). H&E: hematoxylin and eosin

The histopathological examination showed a tumor composed of pleomorphic spindle-shaped to stellate cells arranged in short fascicles. Cells were infiltrating diffusely, with interspersed large areas of collagenization (Figure 3).

**Figure 4:**
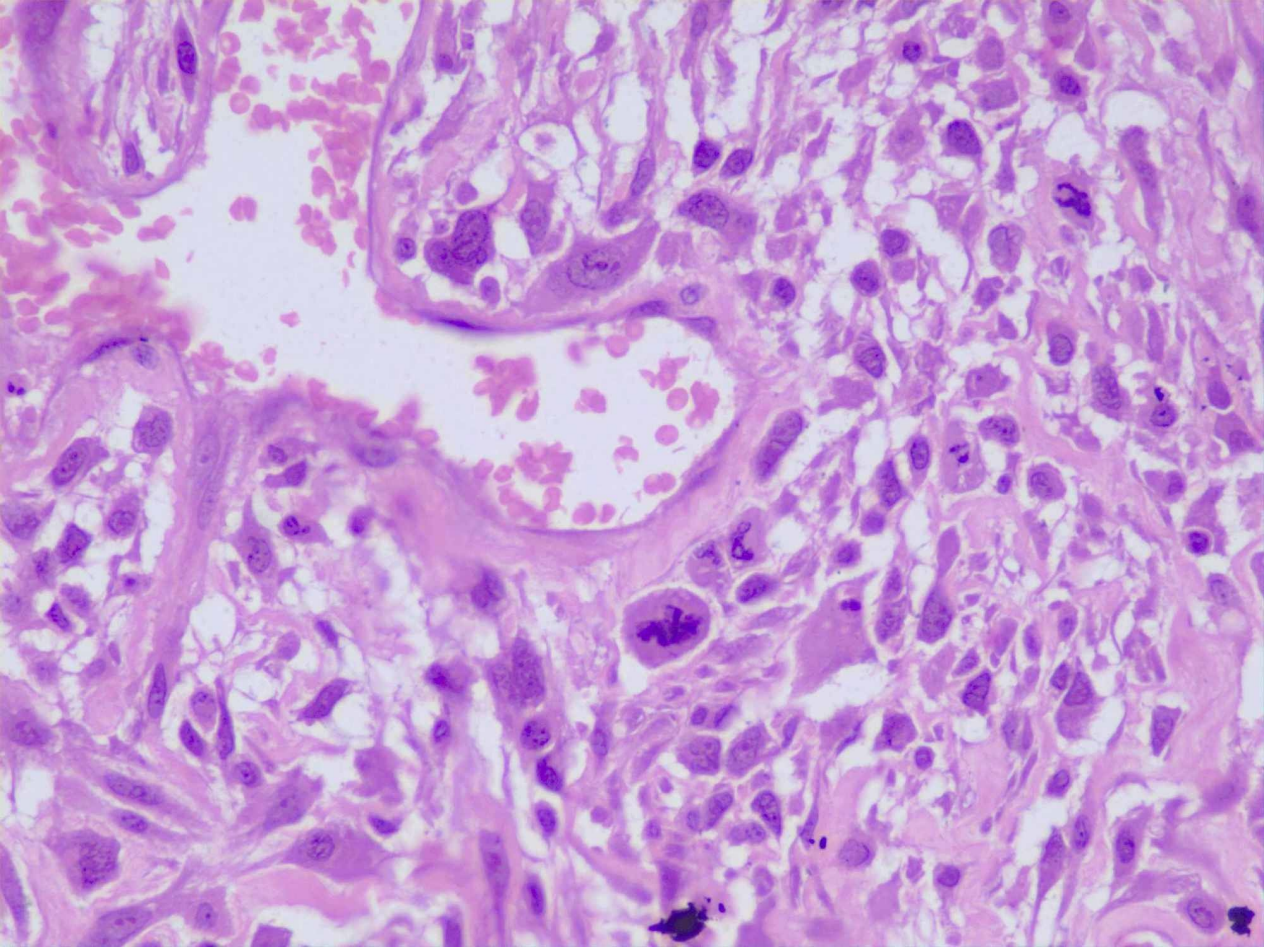
Histopathological examination high power High-power view showing the moderately pleomorphic spindle to stellate-shaped tumor cells with hyperchromatic nuclei and zero to one conspicuous nucleolus. A few mitotic figures are also seen (H&E, 40X). H&E: hematoxylin and eosin

The cells contained moderate amounts of eosinophilic cytoplasm and elongated hyperchromatic nucleus with zero to one conspicuous nucleolus. There were occasional bizarre cells (Figure 4). The mitotic index was a 3/10 high-power field, with a microscope field diameter of 0.65 mm.

**Figure 5:**
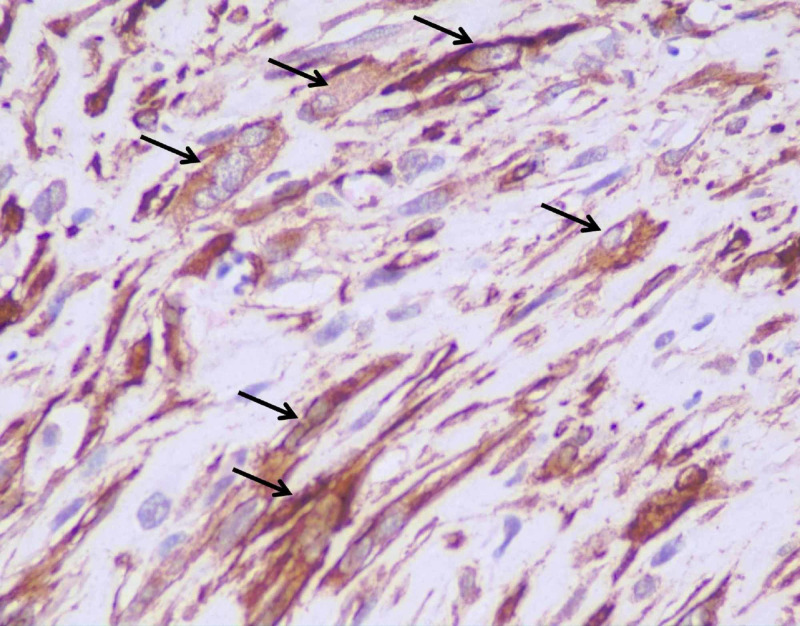
Immunohistochemistry vimentin High-power image showing vimentin positivity in the tumor cells (black arrows) (Vimentin 40X)

**Figure 6:**
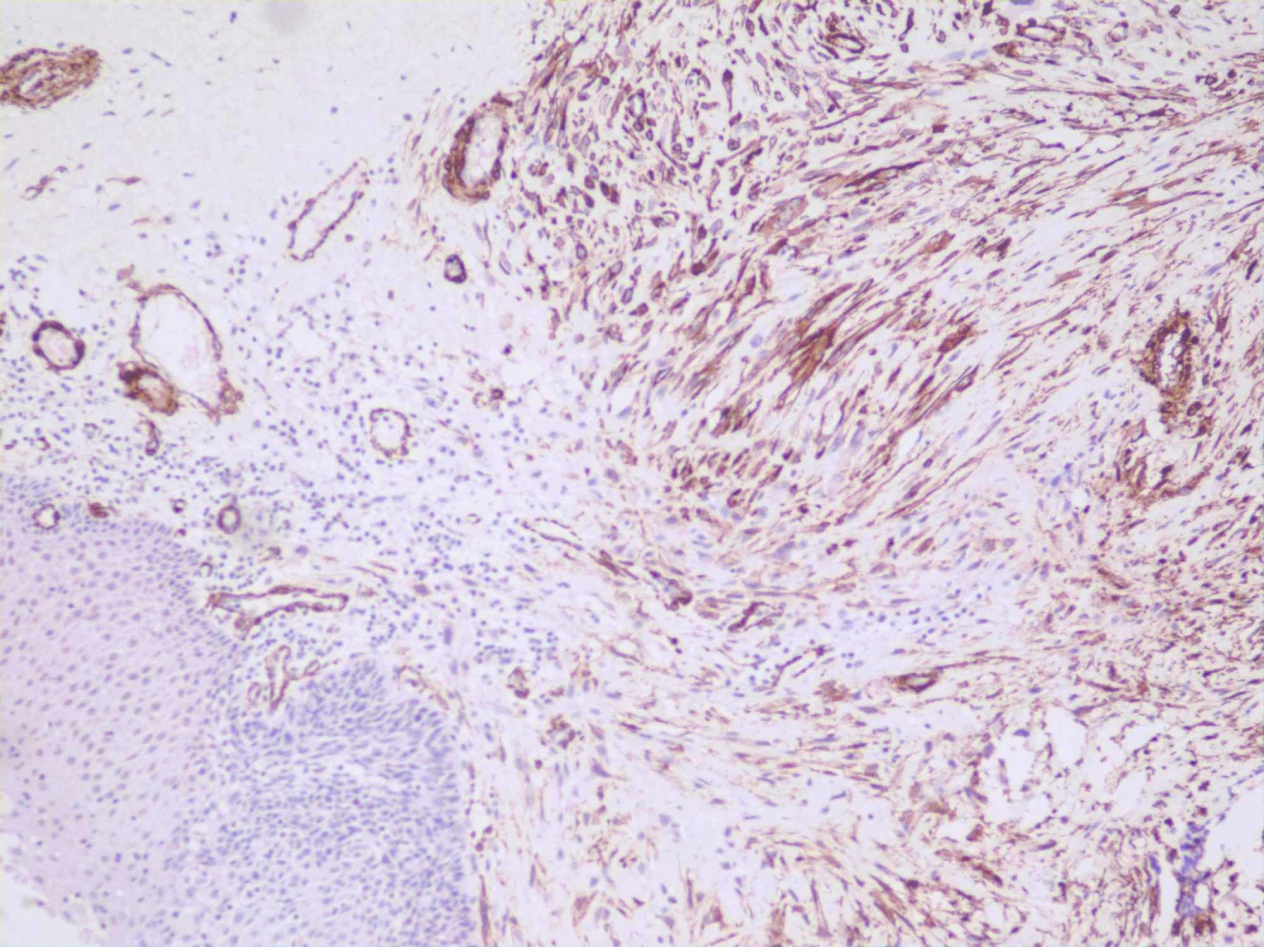
Immunohistochemistry alpha-SMA The tumor cells showing positivity for alpha-smooth muscle antigen (alpha-SMA). The image shows the epithelium on the left side and the underlying tumor on the right side (SMA, 10x).

**Figure 7:**
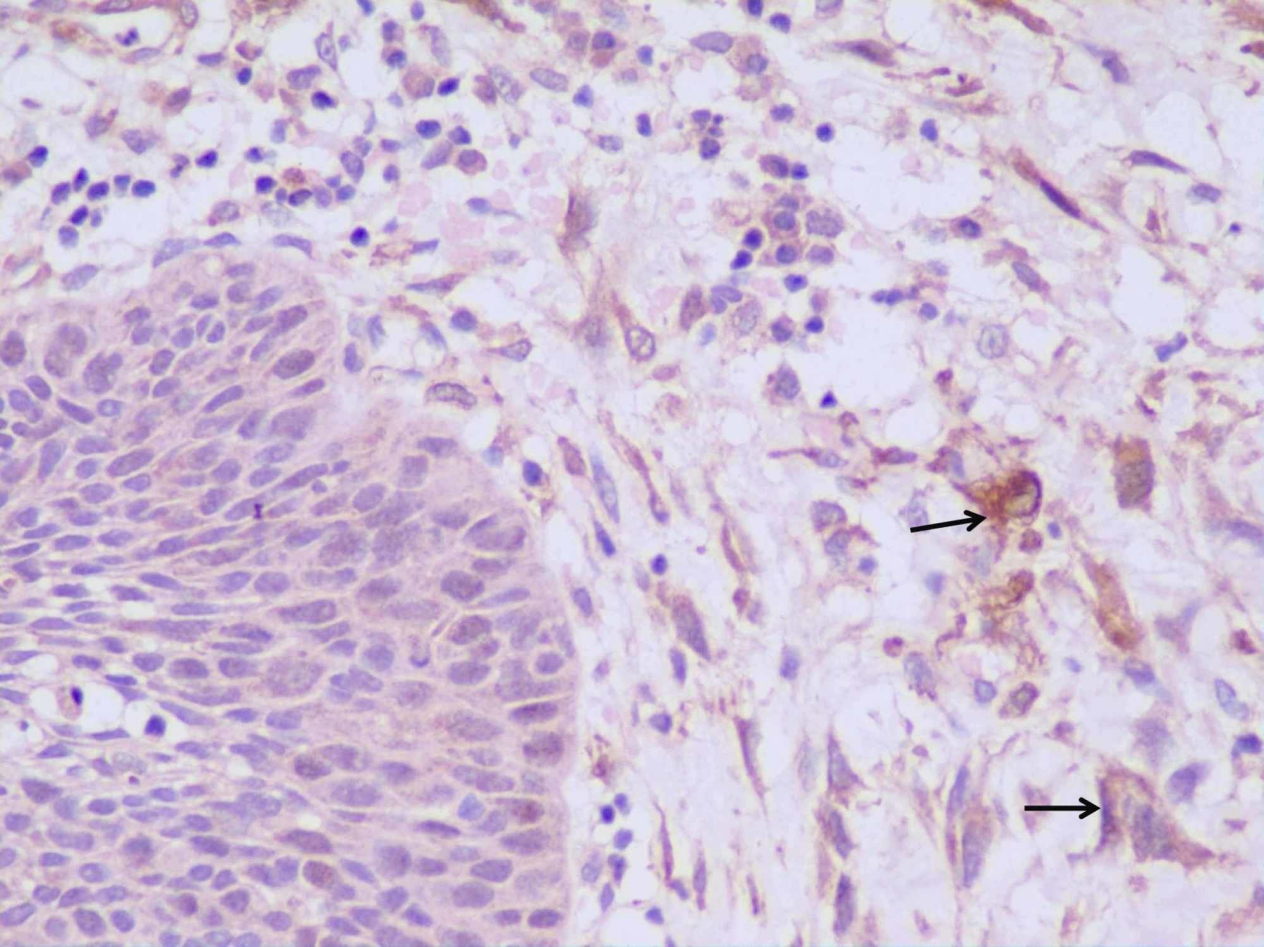
Immunohistochemistry calponin The tumor cells showed positivity for calponin. The image shows the epithelium on the left side and the underlying tumor on the right side (tumor cells arrowed). (Calponin 40X)

There was no lymphovascular invasion. On immunohistochemistry, the tumor cells were positive for vimentin (Figure 5), smooth muscle antigen (SMA) (Figure 6), and focally positive for calponin (Figure 7).

The tumor cells were negative for cytokeratin (CK), epithelial membrane antigen (EMA), cluster of differentiation (CD34), desmin, and myogenin. The IHC was suggestive of low-grade myofibroblastic sarcoma. All the margins were free of tumor.

The patient has been kept on regular follow-up. No adjuvant therapy was given. The patient was offered the cafeteria approach for different voice rehabilitation techniques. Considering the recurrent cost for a tracheoesophageal prosthesis, he preferred to use an electrolarynx for voice rehabilitation. The patient is doing well for two years.

## Discussion

Myofibroblasts are spindle cells that combine the features of both fibroblasts and smooth muscle cells. They have an essential role in wound healing, the inflammation process, and organ remodeling. Histologically, myofibroblasts appear as large spindle-shaped to stellate cells. These cells have round to oval nuclei with deep indentations and conspicuous nucleoli. They are characterized by acidophilic and fibrillary appearing cytoplasm with long cytoplasmic extensions.

Fibrosarcoma, leiomyosarcoma, spindle cell carcinoma, and benign mesenchymal spindle cell lesion-like fibrous histiocytoma are some of the differential diagnoses. Inflammatory myofibroblastic sarcoma looks clinically suspicious, and histologically, it mimics squamous cell carcinoma. Eighty percent of people have the disease on their vocal fold [6].

Myofibroblastic spindle cells with mature lymphocytes, histiocytes, and plasma cells may suggest myofibroblastic tumors. Histological patterns like fascitis-like, compact spindle cell, and hypocellular fibrous patterns are often characteristics of these tumors. Mongomery et al. studied a series of myofibrosarcoma where all cases had fascicular or storiform patterns [7]. Immunohistochemistry, along with a histopathological examination, will aid in diagnosis [8]. In our case, the tumor was positive for vimentin, SMA, and calponin.

Surgery is the mainstay of treatment in these cases. As the lesion was involving the subglottis and thyroid cartilage, total thyroidectomy was done along with total laryngectomy. As the tumor was a low-grade tumor and all margins were free of tumor, no adjuvant therapy was considered. In N0 neck, central compartment clearance should be done along with the excision of the main specimen. Covello et al. reported a patient where they achieved complete surgical clearance (supracricoid laryngectomy with cricohyoidoepiglottopexy). The patient was disease-free for one year, even though they didn't give any adjuvant therapy [9]. In another series of 38 patients by Friedman et al., all patients were operated on with endoscopic resection and followed up [10]. In a case reported by Khosla D et al., the patient underwent total laryngectomy, partial pharyngectomy, and total thyroidectomy. This patient was considered for postoperative radiotherapy because of margin involvement [11]. In our case, as the margins were free, we did not give any adjuvant therapy.

As the tumor has a slow growth rate, these lesions recur late. The recurrent lesions, histologically, do not show a proliferative activity or increase in atypia. A laryngeal preservation resection with clear margins has been reported as oncologically adequate [10]. But still, the evidence is inadequate currently to consider conservative laryngectomy as the treatment of choice. Patient compliance is also a critical factor in deciding management. The patient has to be compliant on regular follow-up for adequate surveillance. Chemoradiation may be considered for unresectable tumors [12].

There are no studies that have discussed long-term survival, as the majority of the studies comment on one-year survival. The risk of local recurrence and metastasis should mandate long-term observation. A high mitotic index, tumor size of more than 10 cm in size, tumor necrosis, and deep location may be considered as a risk factor for metastasis.

## Conclusions

Laryngeal low-grade myofibroblastic sarcoma, even though rare, should be considered a differential diagnosis along with other laryngeal tumors. Clinically, it may mimic malignant lesions or even a papillomatous lesion. These tumors have a good prognosis as compared to other malignancies. Histopathological examination and Immunohistochemistry are key to the diagnosis of this rare tumor. Surgery is the treatment of choice if complete resection is possible. Adjuvant therapy may be considered in case margins are positive. More case series are needed to identify the nature, prognosis, and treatment options of these rare tumors.
